# N-Terminus-Mediated Degradation of ACS7 Is Negatively Regulated by Senescence Signaling to Allow Optimal Ethylene Production during Leaf Development in *Arabidopsis*

**DOI:** 10.3389/fpls.2017.02066

**Published:** 2017-12-06

**Authors:** Gongling Sun, Yuanyuan Mei, Dewen Deng, Li Xiong, Lifang Sun, Xiyu Zhang, Zewen Wen, Sheng Liu, Xiang You, Nasrullah, Dan Wang, Ning Ning Wang

**Affiliations:** Department of Plant Biology and Ecology, College of Life Sciences, Nankai University, Tianjin, China

**Keywords:** leaf senescence, ethylene, ACS7, signal transduction, post-transcriptional regulation

## Abstract

Senescence is the final phase of leaf development, characterized by key processes by which resources trapped in deteriorating leaves are degraded and recycled to sustain the growth of newly formed organs. As the gaseous hormone ethylene exerts a profound effect on the progression of leaf senescence, both the optimal timing and amount of its biosynthesis are essential for controlled leaf development. The rate-limiting enzyme that controls ethylene synthesis in higher plants is ACC synthase (ACS). In this study, we evaluated the production of ethylene and revealed an up-regulation of *ACS7* during leaf senescence in *Arabidopsis*. We further showed that the promoter activity of *ACS7* was maintained at a relatively high level throughout the whole rosette development process. However, the accumulation level of ACS7 protein was extremely low in the light-grown young seedlings, and it was gradually restored as plants aging. We previously demonstrated that degradation of ACS7 is regulated by its first 14 N-terminal residues, here we compared the phenotypes of transgenic *Arabidopsis* overexpressing a truncated ACS7 lacking the 14 residues with transgenic plants overexpressing the full-length protein. Results showed that seedlings overexpressing the truncated *ACS7* exhibited a senescence phenotype much earlier than their counterparts overexpressing the full-length gene. Fusion of the 14 residues to SSPP, a PP2C-type senescence-suppressed protein phosphatase, effectively rescued the *SSPP*-induced suppression of rosette growth and development but had no effect on the delayed senescence. This observation further supported that N-terminus-mediated degradation of ACS7 is negatively regulated by leaf senescence signaling. All results of this study therefore suggest that ACS7 is one of the major contributors to the synthesis of ‘senescence ethylene’. And more importantly, the N-terminal 14 residue-mediated degradation of this protein is highly regulated by senescence signaling to enable plants to produce the appropriate levels of ethylene required.

## Introduction

Senescence is the last phase of leaf development characterized by the mobilization and recycling of a great majority of nutrients to newly formed organs (Lohman et al., 1994; Himelblau and Amasino, 2001). This process is highly regulated and must be appropriately integrated into whole-plant developmental processes. For this reason, senescence mutants with precocious or delayed senescence are often characterized by abnormal growth and seed development (Uauy et al., 2006; Xu et al., 2011; Wu et al., 2012). Although the optimal timing of initiation and progression of leaf senescence are prerequisites for controlled plant growth and development, the mechanisms underlying these processes remain largely unknown.

The gaseous phytohormone ethylene is a widely acknowledged positive regulator of leaf senescence. The progression of this developmental process can therefore be significantly altered by manipulating biosynthesis or signal transduction of ethylene. Both the exogenous ethylene treatment (Zacarias and Reid, 1990) and over-expression of genes encoding key components like EIN3 in ethylene signaling pathway (Li et al., 2013) induce early senescence in *Arabidopsis*. When ethylene biosynthesis or signaling is blocked, for example via antisense suppression of the ACC oxidase in tomato or mutations of the ethylene receptor ETR1 in *Arabidopsis*, delayed leaf senescence can be induced (John et al., 1995; Resnick et al., 2006). We have previously shown that the leucine-rich repeat-receptor-like kinase AtSARK acts as a positive regulator of leaf senescence in *Arabidopsis*. Overexpression of this kinase led to significant induction of both ethylene biosynthesis and subsequent responses. At the same time, inhibition of ethylene biosynthesis alleviated the senescence induced by AtSARK while mutation of the key ethylene signaling component EIN2 completely reversed AtSARK-mediated senescence (Xu et al., 2011). We have also shown that overexpression of the PP2C protein phosphatase-encoding gene *SSPP*, an interactor and negative regulator of *AtSARK* in *Arabidopsis*, resulted in a significant suppression of ethylene biosynthesis and responses, while senescence was greatly delayed in the *SSPP*-overexpressing transgenic plants (Xiao et al., 2015). These observations suggest that both optimal timings and levels of ethylene production are required to control leaf development.

The ethylene biosynthetic pathway in higher plants has been well-documented (Yang and Hoffman, 1984); the rate-limiting step is known to be the conversion of S-adenosylmethionine (SAM) to 1-aminocyclopropane-1-carboxylic acid (ACC) catalyzed by ACC synthase (ACS). Regulation of this enzyme is therefore essential for controlling the rate and level of ethylene production. In most higher plants, ACS is encoded by a multigene family. Based on their C-terminal sequences, ACS proteins can be divided into three main groups. The type 3 ACSs possess a very short C-terminal extension that lacks the conserved sites for CDPK or MAPK6 phosphorylation found in types 1 and 2 ACS proteins. There are nine *ACS* genes in *Arabidopsis* of which ACS7 is the only type 3 ACS (Yamagami et al., 2003).

To fulfill the requirement for stringent regulation of ethylene biosynthesis, the expression of *ACS* members is highly synchronized at both transcriptional and post-transcriptional levels. At the transcriptional level, distinct *ACS* subsets are expressed in response to different developmental, environmental and hormonal signals (Tsuchisaka and Theologis, 2004; Wang et al., 2005). Ethylene biosynthesis is also tightly controlled by both autocatalytic and autoinhibitive regulations (Kende, 1993). We have previously shown that the transcription of *ACS7* is significantly enhanced via exogenous ethylene application and can be markedly suppressed in the ethylene receptor mutant *etr1-1* (Wang et al., 2005; Tang et al., 2008).

At the post-transcriptional level, stability of type 1 ACS isoforms can be enhanced via phosphorylation by either MAPKs or CPKs (Liu and Zhang, 2004; Han et al., 2010; Luo et al., 2014) while negatively affected via dephosphorylations by protein phosphatases 2A and 2C, respectively (Skottke et al., 2011; Ludwików et al., 2014). Type 2 ACS proteins have a unique C-terminal *cis*-acting sequence called TOE domain that is recognized specifically by ETO1/EOL1/EOL2 proteins in ubiquitin-dependent proteolysis (Yoshida et al., 2006; Prasad et al., 2010). It is also possible that ACS proteins of this type can be phosphorylated on the CDPK motif although the detailed mechanisms of this process remain unclear (Hernández Sebastià et al., 2004). At the same time, ACS7, the only type 3 ACS in *Arabidopsis* that lacks C-terminal regulation sites, can also be degraded via the ubiquitin-26S proteasome pathway that requires XBAT32 E3 ligases (Lyzenga et al., 2012). We have recently demonstrated that the 1–14 residues on its N-terminus are involved in the post-translational regulation of ACS7 (Xiong et al., 2014). In addition, ACS proteins are also known to form homo- and hetero-dimers, 25 of which exhibit catalytic activity and render the post-transcriptional regulation of this gene family even more sophisticated (Tsuchisaka and Theologis, 2004). Taken together, all these regulatory mechanisms enable the optimal ethylene production required by different tissues, developmental stages and environmental conditions.

Great achievements so far have been made on elucidating the mechanisms underlying transcriptional and post-transcriptional regulations of *ACS* genes. However, few studies aimed to determine the specific members of this gene family that contribute significantly to the ethylene biosynthesis during leaf senescence, let alone to unravel the underlying mechanisms on how the expressions of *ACS* members are coordinated at both transcriptional and post-transcriptional levels to meet the requirement of plant development and senescence progression. Here we demonstrated that ACS7 is a major contributor to ethylene production during leaf senescence in *Arabidopsis*. And the results of this study also show that the N-terminus-mediated ACS7 degradation is highly regulated by senescence signals to enable optimal ethylene production at the appropriate time during leaf development.

## Materials and Methods

### Plant Materials and Growth Conditions

*Arabidopsis thaliana* ecotype Columbia-0 was used throughout the study apart from the mutant assay where *Arabidopsis* ecotype Wassilewskija-4 was used for both the wild-type and the T-DNA insertion line of *ACS7*. The sources of the two materials in the latter case were described in Dong et al. (2011). The T-DNA insertion line of *ACS7* was further confirmed to have a single insertion site by southern blot analysis (**Supplementary Figure S1**) in this study following standard protocols (Sambrook and Russell, 2001). The generation of transgenic *Arabidopsis GVG:ACS7*^Δ*1*-*14*^-*Flag, GVG:ACS7-Flag, ACS7:GUS* and *ACS7:N*^*7*(*1*-*54*)^-*GUS* were described in Xiong et al. (2014). In order to generate *35S:ACS7-eGFP* and *35S:ACS7*^Δ*1*-*14*^-*eGFP, ACS7* and *ACS7*^Δ*1*-*14*^ were inserted into the binary vector pCABMBIA1301 and fused with a *GFP* gene to create the recombinant transcription units, *ACS7-eGFP* and *ACS7*^Δ*1*-*14*^-*eGFP*, respectively. Similarly, in order to generate *35S:N*^*7*(*1*-*14*)^-*SSPP-HA*, a fusion construct was obtained via PCR by initially using the *35S-N7-SSPP-F2* and *35S-N7-SSPP-R2* primer pair and the *SSPP* gene (Xiao et al., 2015) as template. The diluted PCR product was then used as a template while *35S-N7-SSPP-F1* and *35S-N7-SSPP-R1* were used as the second primer pair to accomplish construction. Constructions of this fusion gene have been patented by our group in the State Intellectual Property Office of the People’s Republic of China with number 201510050322.1 and we have also applied for the international patent with application number PCT/CN2016/070504. The fusion gene was subsequently transferred into the pBI121 expression vector. The construction of *SSPP-HA* was generated on the basis of *N*^*7*(*1*-*14*)^-*SSPP-HA* by removing the *N*^*7*(*1*-*14*)^ part through double digestion. All the primer sequences used in this study are listed in Supplementary Table S1. All recombinant constructs were transferred into *Arabidopsis* by *Agrobacteria*-mediated floral dip method (Clough and Bent, 1998). Recombinant constructs in transgenic plants were confirmed by PCR genotyping and homozygous plants were used for experiments.

Seeds were surface sterilized in 10% (v/v) sodium hypochlorite (Tianjin Chemicals, 559) for 2 min, washed at least 10 times with sterilized water and geminated on one-half-strength Murashige Skoog (MS) medium (Duchefa Biochemie, M0222) containing 0.8% (w/v) agar (Solarbio, A8190), pH5.7, 1% (w/v) Suc (Jiangtian Chemicals, 11411), supplemented with or without antibiotics, stratified at 4°C for 2 days in the dark, and then grown in plant growth chamber at 22/19°C with cycles of 16 h light and 8 h darkness under 100 to 150 μmol m^-2^ s^-1^ light intensity. The 10-day-old seedlings were then transferred to soil and grown under the same conditions for further experiments. Where dexamethasone (DEX, Sigma, D1756) spray was applied, the compound was dissolved in ethanol and the final concentration was at 30 μM.

### Measurements of Ethylene Emission and Chlorophyll Content

Ethylene emission of the 41-day-old wild-type *Arabidopsis* was determined in 10 different leaves of one rosette by gas chromatography (Agilent 7890A) as described in Li and Mattoo (1994). Approximately two leaves of each were separately collected depending on their sizes and vial spacing. Detached leaves were subsequently incubated in vials under light before analysis. The chlorophyll content in mesophyll cells was spectrophotometrically measured as described in Arnon (1949). At least six independent biological replicates were carried out.

### Histochemical GUS Staining

GUS (β-glucuronidase) histochemical staining of transgenic *Arabidopsis* was carried out as described previously in Wang et al. (2005). The images of blue-colored whole plants were recorded with a scanner (Epson V30). Three to six biological replicates for each transgenic line were performed in this study. In total five and seven independent transgenic lines were generated for *ACS7:GUS* and *ACS7:N*^*7*(*1*-*54*)^-*GUS*, respectively, and data shown were the typical results of lines with approximately same levels of *GUS* gene expression.

### RNA Extraction and RT-PCR Analysis of Gene Expression

RNA extraction, cDNA synthesis and RT-PCR analysis were performed as described previously in Liu et al. (2010). The real-time RT-PCR was done using SYBR Green Perfect Mix (Tiangen, FP205-02) on iQ5 (Bio-Rad) machine following the manufacturer’s instructions. All reactions were performed under the following conditions: 95°C for 2 min, 40 cycles of 95°C for 10 s, and 56°C or 58°C in case of *SSPP* amplification for 30 s. *TIP41-like* gene was used as an internal control. The calculation of relative gene expression levels was as described in Xiong et al. (2014). At least three independent replicates were performed to give typical results shown here. All primers used in RT-PCR analysis were listed in Supplementary Table S1.

### Protein Extraction and Immunoblot Analysis

Seedling or leaf samples were ground in liquid nitrogen and total soluble protein was extracted as described in Wang et al. (2005). The concentration of protein extract was measured using the Bradford reagent (USB, DH078) and total protein was separated by 12% SDS-PAGE gel and detected by antibodies after being transferred to a polyvinylidene fluoride (PVDF) membrane (GE Healthcare, 10600023). The antibodies used in this study include rabbit anti-GFP (Abcam, ab290), mouse anti-Flag (Sigma, F3165), rabbit anti-HA (Abcam, ab9110), mouse anti-actin (Abmart, 26F7), mouse anti-Tubulin (Abmart, 10B1), goat anti-rabbit IgG H&L (HRP) (Abcam, ab6721) and rabbit anti-mouse IgG H&L (HRP) (Abcam, ab6728). Protein bands were visualized using an ECL western blotting reagent pack (GE Healthcare, RPN2232) flowing the manufacturer’s instructions. Images were recorded by a chemiluminescence imaging system (CELVIN’s, Biostep). Quantification of the signals was performed in Image J software as described in Xiong et al. (2014).

## Results

### The Peak of Ethylene Emission Occurs after the Initiation of Leaf Senescence

**FIGURE 1:**
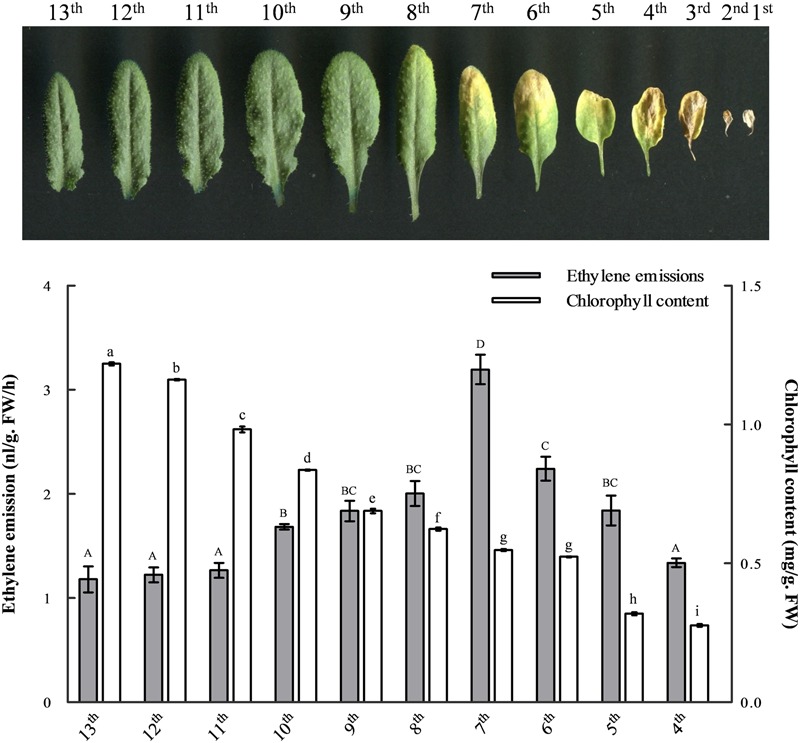
The changes of ethylene emission and chlorophyll content during leaf development. Leaves from 41-day-old wild-type *Arabidopsis* were laid out in the reverse order of emergence. The ethylene emission and chlorophyll content in developing leaves were measured, respectively, for each leaf from the 13th to the fourth. At least six independent biological replicates were performed. Different letters indicate statistically significant differences based on analysis of variance (ANOVA) (α = 0.05) using upper case for ethylene and lower case for chlorophyll measurements. Error bars represent standard errors (SE).

To evaluate the spatiotemporal distribution of ethylene biosynthesis and the correlations between ethylene production and leaf senescence, we measured changes in ethylene emissions and chlorophyll contents in the developing leaves of 41-day-old *Arabidopsis* (**Figure 1**). The results showed that the content of chlorophyll in mesophyll cells was gradually decreased during leaf senescence and the most significant decline was between leaves ten and nine. At the same time, however, the level of ethylene production was gradually increased from the mature 10th leaf, reaching a peak in the seventh. In the older leaves, from the sixth to the fourth, both the chlorophyll content and ethylene emission were continuously dropped (**Figure 1**). Indeed, both chlorophyll content and ethylene production were hardly measurable in the much more senescent leaves, including the third, second and first (data not shown).

### The Expression of *ACS7* Is Up-regulated in Both Natural and Dark-Induced Leaf Senescence Processes

**FIGURE 2:**
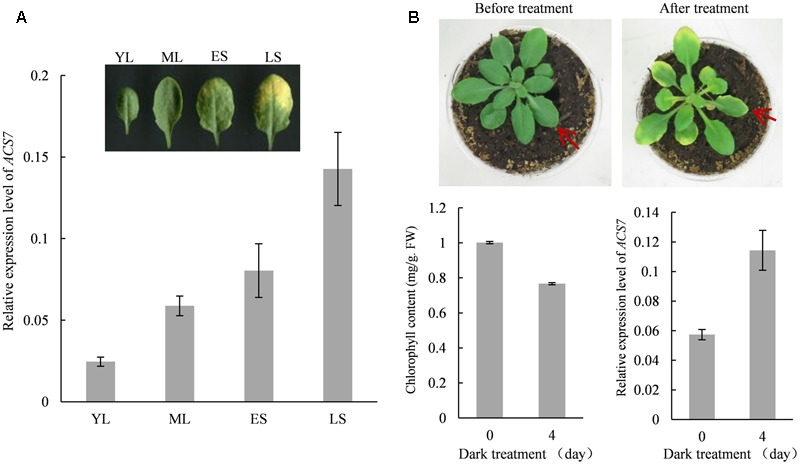
The expression of *ACS7* is up-regulated during natural and dark-induced leaf senescence. **(A)** Quantitative RT-PCR analysis of the transcription levels of *ACS7* in developing leaves. YL: a young leaf with half the size of a fully expanded leaf. ML: a fully expanded, non-senescent mature leaf. ES: an early senescent leaf with less than 25% leaf area yellowing. LS: a late senescent leaf with more than 50% leaf area yellowing. **(B)** Phenotypic presentation and measurements of chlorophyll content and transcription levels of *ACS7* in wild-type *Arabidopsis* at stage 5.10 before and after dark-treatment. RNA was extracted from the seventh leaf and quantitative RT-PCR was performed using *TIP41-like* as internal control. The experiments were repeated three times with similar results. Error bars represent standard deviations (SD).

The quantitative RT-PCR assay was performed to determine the involvement of *ACS7* in *Arabidopsis* leaf senescence. Results showed that during natural senescence, the transcription levels of *ACS7* remained relatively low in young leaves but increased gradually as they developed to late senescence stages (**Figure 2A**). It was also shown that 4-day dark treatment resulted in a significant decrease in the chlorophyll content in the wild-type *Arabidopsis* plants at stage 5.10 (Boyes et al., 2001). In the meantime, however, an obvious increase in the level of *ACS7* transcript was revealed during the dark-induced senescence process (**Figure 2B**).

### High-Level Accumulation of ACS7 Protein Promotes Leaf Senescence in *Arabidopsis*

**FIGURE 3:**
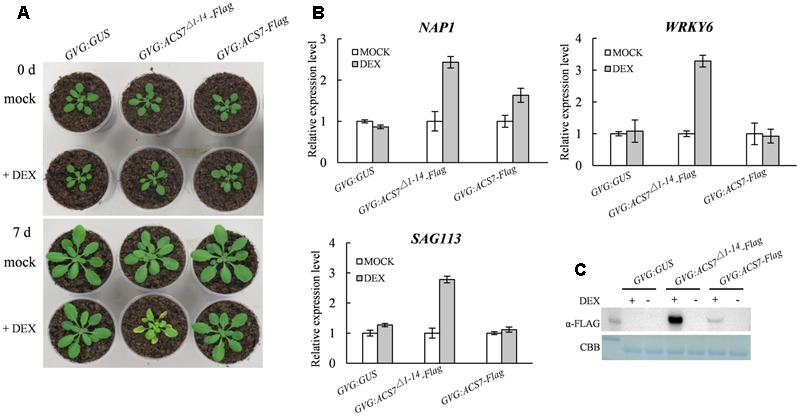
High accumulation of the ACS7 protein promotes leaf senescence in *Arabidopsis.* **(A)** The 25-day-old *GVG:GUS, GVG:ACS7*^Δ*1*-*14*^-*Flag* and *GVG:ACS7-Flag* transgenic *Arabidopsis* plants were sprayed daily with either 30 μM DEX (+DEX) or its solvent, ethanol (+mock), for 3 days. The rosettes were photographed at an additional 4 days. **(B)** The expressions of several senescence-associated marker genes were up-regulated in the leaves of *GVG:ACS7*^Δ*1*-*14*^-*Flag* after dark treatment. The 25-day-old *GVG:GUS, GVG:ACS7*^Δ*1*-*14*^-*Flag* and *GVG:ACS7-Flag* transgenic *Arabidopsis* plants were sprayed with 30 μM DEX and 24 h later the fifth and sixth leaf of each were harvested for RNA extraction. The expression levels of *NAP1, WRKY6*, and *SAG113* were determined by quantitative RT-PCR analysis with *TIP41-like* used as an internal control; and the relative value of each gene under mock treatment were normalized as 1. **(C)** The 25-day-old *GVG:GUS, GVG:ACS7*^Δ*1*-*14*^-*Flag*, and *GVG:ACS7-Flag* transgenic *Arabidopsis* plants were sprayed with either 30 μM DEX (+DEX) or its solvent, ethanol (mock), and the fifth leaves were harvested after 24 h for protein extraction. Accumulation levels of ACS7-Flag and ACS7^Δ1-14^-Flag were detected using immunoblot with Coomassie brilliant blue staining as the loading control. In total three and two independent transgenic lines were obtained for *GVG:ACS7-Flag* and *GVG:ACS7*^Δ*1*-*14*^-*Flag*, respectively, and data shown here were representatives of the typical results from three independent biological replicates. Error bars represent SD in all cases.

As we have previously reported, the N-terminal 1–14 residues of ACS7 negatively regulate the protein stability. Consistently, the truncated form of ACS7 lacking the 14 residues is more stable than the full-length form when transgenically expressed in the light-grown *Arabidopsis* seedlings (Xiong et al., 2014). We therefore used previously generated transgenic *Arabidopsis* plants expressing Flag-tagged full-length ACS7 as well as truncated ACS7 with 1–14 residues deleted under the control of a DEX-inducible promoter, namely *GVG:ACS7-Flag* and *GVG:ACS7*^Δ*1*-*14*^-*Flag* (Xiong et al., 2014) in this study to examine the detailed effects of *ACS7* on leaf senescence. Quantitative RT-PCR revealed comparable transcript levels of *ACS7* in these two transgenic materials (**Supplementary Figure S2**). It was shown that the *GVG:ACS7-Flag* transgenic *Arabidopsis* exhibited normal growth and development compared to the *GVG:GUS* control plants after 4 days following DEX spray. In contrast, the *GVG:ACS7*^Δ*1*-*14*^-*Flag* transgenic plants displayed prominent leaf yellowing and had significantly smaller rosettes (**Figure 3A**), as initially noted by Xiong et al. (2014). We further sampled the fifth and sixth leaves of *GVG:GUS, GVG:ACS7-Flag* and *GVG:ACS7*^Δ*1*-*14*^-*Flag* after DEX treatment and performed quantitative RT-PCR to determine the transcription levels of several known senescence-associated marker genes including two transcriptional factors *NAP1* (Guo and Gan, 2006) and *WRKY6* (Robatzek and Somssich, 2002), as well as one PP2C family protein phosphatase gene *SAG113* (Zhang et al., 2012). No significant increases in the transcription levels of these marker genes were found in the DEX-treated *GVG:GUS* and *GVG:ACS7-Flag* plants while the expressions of these senescence-associated markers in the DEX-treated *Arabidopsis* expressing *ACS7*^Δ*1*-*14*^-*Flag* were greatly up-regulated (**Figure 3B**). Moreover, western blot assay using anti-Flag antibody revealed a much higher level of ACS7 protein accumulation in the fifth leaves of DEX-treated *GVG:ACS7*^Δ*1*-*14*^-*Flag* plants than in their *GVG:ACS7-Flag* counterparts (**Figure 3C**, for original images of the blots please see **Supplementary Figure S6**). These results confirmed that the induced high-level accumulation of ACS7 protein led to precocious leaf senescence in *Arabidopsis*.

### The *acs7-1* Mutant Exhibits Delayed Natural Senescence

**FIGURE 4:**
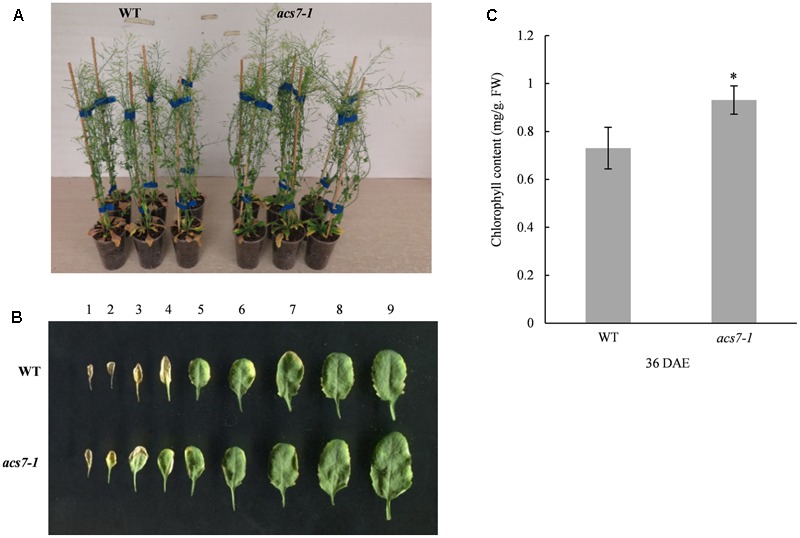
The *acs7-1* mutant showed delayed leaf senescence. **(A)** The *acs7-1* mutant plants and their wild-type control (Wassilewskija-4) were photographed at 44 DAE stage. **(B)** Leaves from *acs7-1* and WT plants at 39 DAE stage were laid out in order of emergence. **(C)** The chlorophyll contents in the fifth and sixth leaves of both *acs7-1* and WT plants were measured at 36 DAE stage. Data represent means ± SD. The experiments were repeated at least three times to give the typical results shown here. Asterisk indicates statistically significant differences in student’s *t*-test (α = 0.01).

In order to further confirm the function of *ACS7* in leaf senescence, we examined the phenotype of a loss-of-function mutant of *ACS7, acs7-1* (Tsuchisaka et al., 2009; Dong et al., 2011). As was shown in **Figures 4A,B**, the *acs7-1* mutant exhibited delayed leaf senescence compared to its wild-type control (Wassilewskija-4). In line with the phenotypic observations, the chlorophyll content in the *acs7-1* mesophyll cells was significantly higher than in the wild-type control when measured at 36 days after emergence (DAE) (**Figure 4C**). The *acs7-1* mutants exhibited larger rosettes, faster growth, and reached greater heights (**Supplementary Figure S3**). We also observed an early flowering phenotype within these mutant plants, consistent with a previous report (Tsuchisaka et al., 2009), as well as a shorter blooming period in comparison to their wild-type control (**Supplementary Figure S3**).

### The Accumulation of ACS7 Protein Is Extremely Low in Light-Grown Young Seedlings but Is Gradually Restored As Plants Aging

**FIGURE 5:**
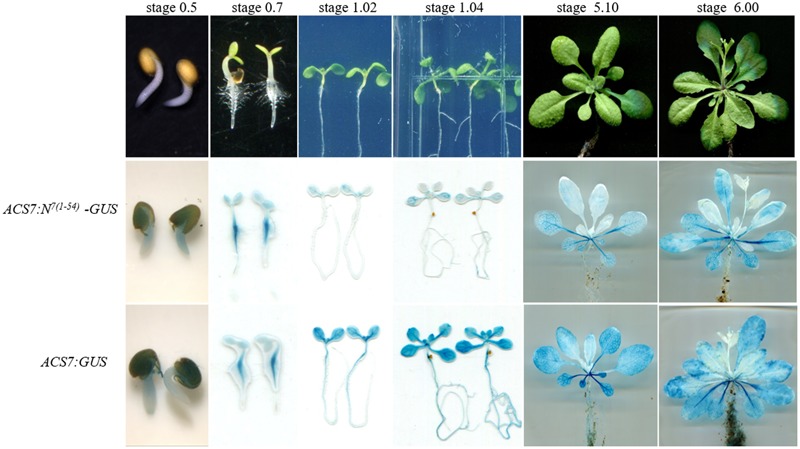
Comparisons of changes in the promoter activity and protein accumulation of ACS7 during leaf development. The light-grown *ACS7:N*^*7*(*1*-*54*)^-*GUS* and *ACS7:GUS* transgenic *Arabidopsis* with approximately the same transcription levels of the *GUS* gene were photographed at six different stages including stage 0.5, 0.7, 1.02, 1.04, 5.10, and 6.00, respectively, as described in Boyes et al. (2001). The GUS staining method was described in section “Materials and Methods.” Three to six independent biological replicates were carried out for each transgenic line to give the typical results presented.

To further explore the underlying mechanisms of *ACS7*-regulated leaf senescence, we generated transgenic *Arabidopsis* expressing the *GUS* gene fused to the N-terminal 1–54 residues of ACS7, namely *N*^*7*(*1*-*54*)^-*GUS*, or a *GUS* gene alone driven by *ACS7* native promoter. Two lines that exhibited no significant differences in levels of transcriptional expression of the *GUS* genes were selected for further study (**Supplementary Figure S4**) and photographed at six different developmental stages including stage 0.5, stage 0.7, stage 1.02, stage 1.04, stage 5.10, and stage 6.00 as previously described by Boyes et al. (2001) (**Figure 5**, upper row). In view of the fact that N-terminal 1–14 residues play an important role in the post-translational regulation of ACS7, the activities of N^7(1-54)^-GUS are thus indicative of changes in the accumulation levels of internal ACS7 protein during leaf development (Xiong et al., 2014). The histochemical GUS staining assay revealed that *ACS7* promoter activity and the accumulation of ACS7 protein were both at very high levels in the late stages of seed germination. With the growth of seedlings, the promoter activity of *ACS7* was maintained at a very high level while its protein accumulation varied from stage to stage (**Figure 5**). For instance, the blue coloring of *ACS7:N*^*7*(*1*-*54*)^-*GUS* was significantly decreased at vegetative stages such as stages 1.02 and 1.04 but was gradually restored at stages 5.10 and 6.00 along with the initiation of leaf senescence. A closer look revealed that the blue colors in *ACS7:N*^*7*(*1*-*54*)^-*GUS* at the last two stages were generally observed in mature and senescent leaves while almost no traces were seen in young leaves (**Figure 5**, middle row). All results presented here suggested that the N-terminal mediated ACS7 degradation was restricted during the process of seed germination, and that the restriction was released at vegetative stages but was tighten again with the progressing of leaf senescence.

### N-Terminus-Mediated ACS7 Protein Degradation Is Negatively Regulated by Senescence Signals

In order to confirm the role of senescence signals in the regulation of ACS7 stability, we generated transgenic *Arabidopsis* expressing GFP-tagged full-length ACS7 (*ACS7-eGFP*) or truncated ACS7 lacking N-terminal 1–14 residues (*ACS7*^Δ*1*-*14*^-*eGFP*) under the control of *35S* promoter.

**FIGURE 6:**
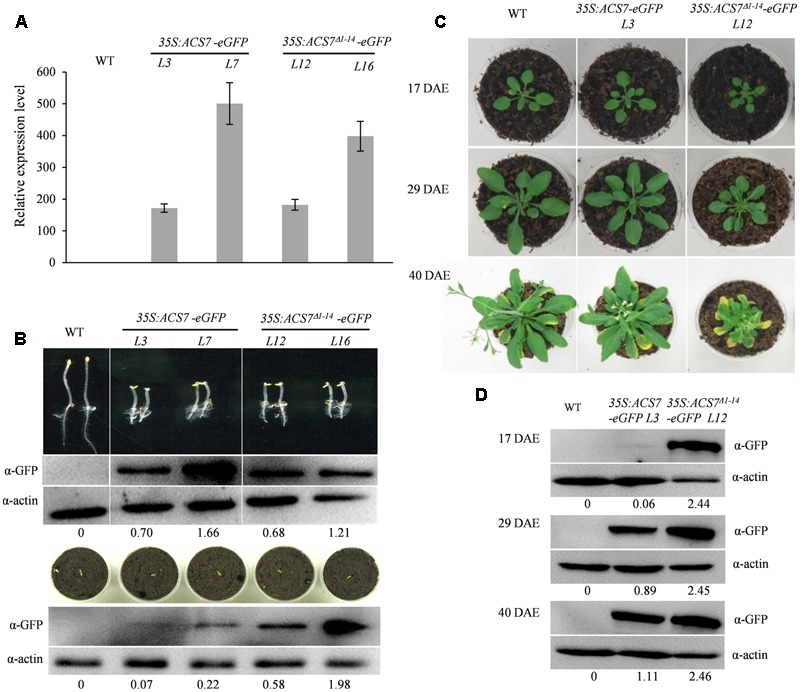
Effects of over-expression of the full-length (*35S:ACS7-eGFP*) or truncated ACS7 (*35S:ACS7*^Δ*1*-*14*^-*eGFP*) on the development of *Arabidopsis* seedlings. **(A)** Quantitative RT-PCR analysis was performed to determine the expression levels of *ACS7* in line 3 and line 7 of *35S:ACS7-eGFP* as well as in line 12 and line 16 of *35S:ACS7*^Δ*1*-*14*^-*eGFP* transgenic *Arabidopsis*. RNA was extracted from 7-day-old seedlings and RT-PCR was performed using *TIP41-like* as an internal control. Data presented by means ± SD were the typical results from three independent biological replicates. **(B)** Phenotypic presentations and ACS7 protein accumulations in etiolated and light-grown seedlings of *35S:ACS7-eGFP* and *35S:ACS7*^Δ*1*-*14*^-*eGFP* transgenic lines. The accumulations of ACS7-eGFP and ACS7^Δ1-14^-eGFP proteins were detected using anti-GFP antibody and the detection of anti-actin served as loading control. Numbers below the blots indicate the intensity ratio of the ACS7 band to the β-actin control band in each lane. **(C)** Young seedlings of the line 3 of *35S:ACS7-eGFP* and line 12 of *35S:ACS7*^Δ*1*-*14*^-*eGFP* as well as their wild-type control at the same developmental stage were transferred from germination plates into soil at 7 DAE and photographed at 17, 29, and 40 DAE, respectively. **(D)** ACS7 protein accumulations in the fifth and sixth leaves of line 3 of *35S:ACS7-eGFP* and line 12 of *35S:ACS7*^Δ*1*-*14*^-*eGFP* transgenic lines were determined at 17, 29, and 40 DAE. Total proteins loaded were 40, 40, and 20 μg for WT, line 3 of *35S:ACS7-eGFP* and line 12 of *35S:ACS7*^Δ*1*-*14*^-*eGFP*, respectively. ACS7-eGFP and ACS7^Δ1-14^-eGFP proteins were detected using anti-GFP antibody and the detection of anti-actin served as loading control. Numbers below the blots indicate the intensity ratio of the ACS7 band to the β-actin control band in each lane. In total six and eight independent transgenic lines were obtained for *35S:ACS7-eGFP* and *35S:ACS7*^Δ*1*-*14*^-*eGFP*, respectively, and data shown here were representatives of the typical results from at least three independent biological replicates.

To begin with, we evaluated expression levels of the *ACS7* transgenes using quantitative RT-PCR. The results showed that the transcript levels of *ACS7* in line 7 of *35S:ACS7-eGFP* and in line 16 of *35S:ACS7*^Δ*1*-*14*^-*eGFP* were about 500 and 400 times higher, respectively, than that of the internal control *TIP41-like*. In line 3 of *35S:ACS7-eGFP* and line 12 of *35S:ACS7*^Δ*1*-*14*^-*eGFP*, the expressions of the transgenes were at approximately the same but relatively much lower levels (**Figure 6A**).

The phenotypic analysis of etiolated seedlings showed that all of the four lines mentioned above exhibited prominent triple responses, implying that expression of *ACS7*^Δ*1*-*14*^-*eGFP* and *ACS7-eGFP* both led to over-production of ethylene at this developmental stage (**Figure 6B**, upper panel). Immunoblot analysis also revealed approximately equally high levels of ACS7 accumulation in the transgenic etiolated seedlings of line 3 of *35S:ACS7-eGFP* and line 12 of *35S:ACS7*^Δ*1*-*14*^-*eGFP*, however, the light-grown line 3 seedlings of *35S:ACS7-eGFP* exhibited a much lower accumulation level of ACS7 protein in comparison to line 12 of *35S:ACS7*^Δ*1*-*14*^-*eGFP* (**Figure 6B**, for original images of the blots please see **Supplementary Figure S6**), even though the transcripts of *ACS7* in both lines were at similar levels (**Figure 6A**). In addition, line 7 of *35S:ACS7-eGFP*, which had extremely high levels of *ACS7* transcript and protein accumulation in etiolated seedlings (**Figure 6B**, upper panel), also displayed a remarkable decrease in the accumulation level of ACS7 protein in light-grown seedlings compared to the line 16 of *35S:ACS7*^Δ*1*-*14*^-*eGFP* (**Figure 6B**, lower panel, for original images of the blots please see **Supplementary Figure S6**). These results implied that the accumulation of full-length ACS7 was inhibited and further supported our previous findings that the truncated ACS7 protein lacking the N-terminal 1–14 residues was more stable than the full-length one in the vigorously growing seedlings under normal condition (Xiong et al., 2014).

To further evaluate the phenotypic differences in leaf senescence, line 12 of *35S:ACS7*^Δ*1*-*14*^-*eGFP* and line 3 of *35S:ACS7-eGFP* that had approximately same levels of *ACS7* transcript as well as wild type *Arabidopsis* were grown under light-grown conditions and photographed at three different stages including 17, 29, and 40 DAE (**Figure 6C**). The results revealed no significant differences between *35S:ACS7-eGFP* and the wild-type control until 40 DAE when the transgenic plants exhibited slightly earlier senescence and smaller rosette, indicating a restoration of ACS7 protein level in late stages. In contrast, transgenic plants overexpressing the truncated ACS7 with the N-terminal 14 residues deleted exhibited a number of significant ethylene response phenotypes including smaller rosette since 17 DAE and much earlier senescence at 40 DAE. In line with this, the accumulation of ACS7 protein in the fifth and sixth leaves of line 3 of *35S:ACS7-eGFP* was at very low level when the plant was at vegetative stages such as 9 and 17 DAE, however, it was increased when the plant approached its first-flower opening stage at 29 DAE and reached a much higher level at 40 DAE as plant aging. By contrast, the ACS7 proteins in the fifth and sixth leaves of line 12 of *35S:ACS7*^Δ*1*-*14*^-*eGFP* were maintained at a high level throughout all these time points and did not show any significant response to senescence signaling (**Figures 6B,D**). All these results demonstrated again that the N-terminus mediated ACS7 degradation was negatively regulated by senescence signals.

### Fusing ACS7 N-Terminal 14 Residues to SSPP Effectively Rescues the *SSPP*-Induced Growth Suppression but Imposes No Effects on the Delayed Senescence

**FIGURE 7:**
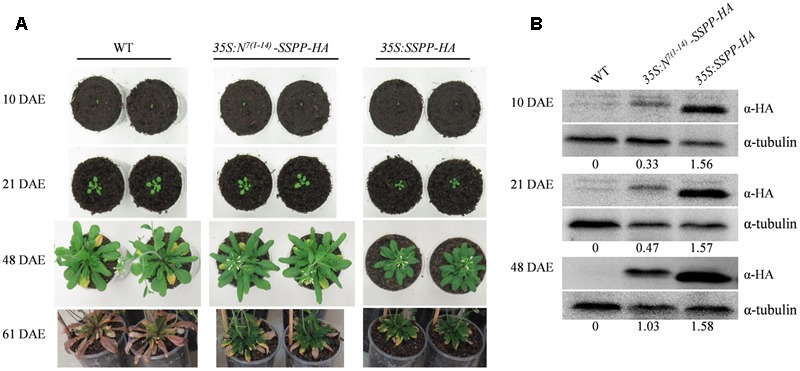
Comparisons of the transgenic *Arabidopsis* over-expressing *SSPP* and *N*^*7*(*1*-*14*)^-*SSPP*. **(A)** The transgenic *Arabidopsis* lines of *35S:SSPP-HA* and *35S:N*^*7*(*1*-*14*)^-*SSPP-HA* as well as their wild-type control at the same developmental stage were transferred from germination plates to soil at 7 DAE and photographed at 10, 21, 48, and 61 DAE, respectively. The experiment was repeated three times with similar results. In total six independent transgenic lines of *35S:N*^*7*(*1*-*14*)^-*SSPP-HA* and four independent transgenic lines of *35S:SSPP-HA* were generated and data shown here were the typical results of each transgenic line. **(B)** SSPP protein accumulations in *35S:N*^*7*(*1*-*14*)^-*SSPP-HA* and *35S:SSPP-HA* at 10, 21, and 48 DAE. SSPP-HA and N^7(1-14)^-SSPP-HA proteins were detected using anti-HA antibody and the detection of tubulin served as loading control. Numbers below the blots denotes the intensity ratio of the SSPP band to the tubulin control band in each lane. At least three independent biological replicates were carried out to give the typical results shown.

**FIGURE 8:**
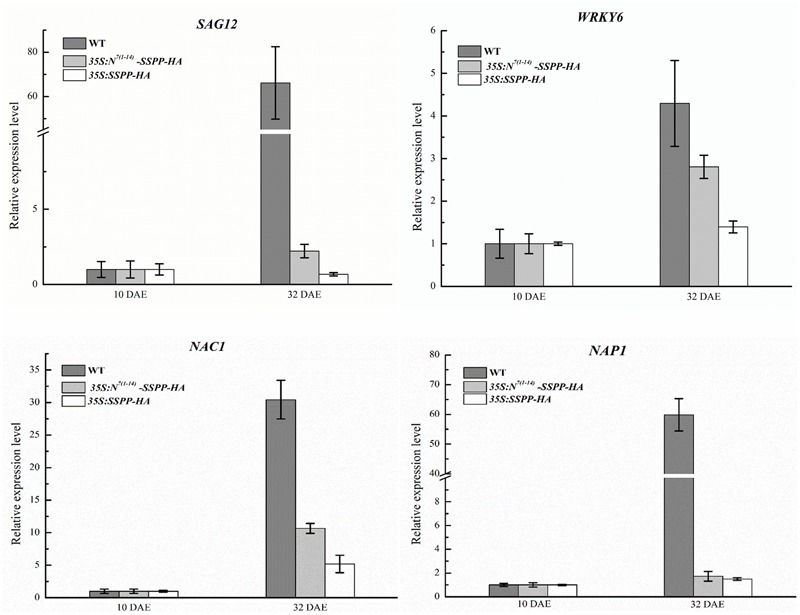
Overexpression of *N*^*7*(*1*-*14*)^-*SSPP* and *SSPP* reduced the expression levels of several critical senescence-associated marker genes in the transgenic *Arabidopsis*. Quantitative RT-PCR analysis was performed to determine the transcription levels of several critical senescence-associated marker genes including *SAG12, WRKY6, NAP1*, and *NAC1* in the *35S:N*^*7*(*1*-*14*)^-*SSPP-HA* and *35S:SSPP-HA* transgenic *Arabidopsis* at 32 DAE. RNA was extracted from the fifth and sixth leaves of each transgenic plant. Bars represent the relative expression levels to internal control gene *TIP41-like* and the relative expression levels of each gene at 10 DAE were normalized as 1. Error bars represent SD.

In order to further confirm the role of senescence signaling in the regulation of N-terminus mediated ACS7 protein degradation, we generated transgenic *Arabidopsis* plants over-expressing HA-tagged SSPP, a negative regulator of *Arabidopsis* leaf senescence, or HA-tagged SSPP fused with the N-terminal 1–14 residues of ACS7 at its N-terminus under the control of *35S* promoters [namely *35S:SSPP-HA* and *35S:N*^*7*(*1*-*14*)^-*SSPP-HA*, respectively]. Two transgenic lines that had approximately the same transcript levels of *SSPP* were used in this study (**Supplementary Figure S5**) and photographed at 10, 21, 48, and 61 DAE, respectively. In line with our previous findings from the *35S:SSPP* plants (Xiao et al., 2015), the *35S:SSPP-HA* transgenic lines displayed a remarkable decrease in rosette size. However, all the *35S:N*^*7*(*1*-*14*)^-*SSPP-HA* transgenic plants developed normally before the onset of leaf senescence, regardless of the similar transcript levels of *SSPP* (**Figure 7A**). And more interestingly, after the initiation of leaf senescence, the *35S:N*^*7*(*1*-*14*)^-*SSPP-HA* transgenic plants also exhibited significantly delayed leaf senescence in comparison to the wild-type control at 48 DAE and much later stages such as 61 DAE (**Figure 7A**). We further carried out western blot assay to evaluate SSPP accumulations in both *35S:N*^*7*(*1*-*14*)^-*SSPP-HA* and *35S:SSPP-HA* transgenic plants at different developmental stages. As expected, while the accumulation levels of SSPP in the *35S:SSPP-HA* plants were constantly maintained at a relatively high and stable level from 10 to 48 DAE stages, the SSPP accumulation in the *35S:N*^*7*(*1*-*14*)^-*SSPP-HA* seedlings was very low at 10 DAE, but it was increased at 21 DAE and reached a higher level at 48 DAE as plants aging (**Figure 7B**). These observations suggested that the N-terminus-mediated degradation of SSPP was also suppressed by senescence signaling. The senescence-delayed effect of *N*^*7*(*1*-*14*)^-*SSPP* fusion gene was further confirmed by the quantitative analysis of several senescence-associated marker genes. As shown in **Figure 8**, the expressions of *SAG12, WRKY6, NAP1*, and *NAC1* were significantly down-regulated in both *35S:N*^*7*(*1*-*14*)^-*SSPP-HA* and *35S:SSPP-HA* transgenic plants in comparison to WT at 32 DAE.

## Discussion

Both the onset and progression of leaf senescence are tightly regulated by genetic programming in order to ensure an adequate supply of assimilative products required for plant growth and development and, at the same time, guarantee a continuous and effective export of nutrients to newly formed organs. As an important senescence-promoting phytohormone, the biosynthesis of ethylene is tightly regulated during leaf development. Jing et al. (2005) noted that the ability of ethylene to induce leaf senescence is dependent on age-related changes. Our results also show that ethylene production in the rosette leaves of *Arabidopsis* was mostly maintained at a relatively low level and the peak of ethylene emission occurred only after the initiation of leaf senescence (**Figure 1**). In addition, we observed continuous decreases in both ethylene and chlorophyll contents in much older leaves from the sixth to the fourth. This is in line with the previous idea that ethylene is needed for chlorophyll breakdown and nutrient remobilization at relatively earlier time points during senescence, but not for the cell death that occurs at the final stage of senescence, which is more under the control of salicylic acid (Morris et al., 2000; Breeze et al., 2011). Nevertheless, how plants coordinate their ethylene biosynthesis with leaf senescence remains an area for further work. Given the number of *ACS* genes present in *Arabidopsis* genome, it will also be essential to determine which members specifically play major roles in the biosynthesis of ‘senescence ethylene’ and elucidate the underlying mechanisms.

In higher plants, the expression and activities of *ACS* family members can be highly regulated at both transcriptional and post-transcriptional levels to enable the optimal levels of ethylene production required in different organs and tissues at distinctive developmental stages. Given the fact that the peak of ethylene production occurred in the later stage of leaf senescence (**Figure 1**) and the biosynthesis of ethylene can be both auto-catalyzed and auto-inhibited (Wang et al., 2005), it can be speculated that the key *ACS* genes that play major roles in ethylene biosynthesis during leaf senescence, namely ‘senescence *ACSs*,’ should all share the following characteristics. In the first place, these genes should be up-regulated at both transcriptional and post-transcriptional levels by senescence signals and auto-catalyzed by ethylene itself. Secondly, the regulations of these genes should correspond with the senescence progress to ensure that the level of ethylene production remains relatively low at vegetative stages but can be further enhanced by the positive feedback mechanism in later stages.

We previously found that the promoter activity of *ACS7* could be positively regulated by ethylene (Wang et al., 2005). And in *AtSARK*-mediated leaf senescence, the expression levels of ACS members that formed active dimers with ACS7, including ACS4, ACS6, ACS9, and ACS7 itself, were all up-regulated, whereas those of the partners that formed inactive dimers such as ACS2, ACS5, and ACS11, were down-regulated (Xu et al., 2011). All these implied that ACS7 may play important roles in the biosynthesis of ‘senescence ethylene’. In the present study, we demonstrated that expression of *ACS7* was up-regulated during both natural and dark-induced leaf senescence (**Figure 2**). Consistently, we further showed that high accumulation of ACS7 protein led to precocious leaf senescence as well as greatly up-regulated expressions of several critical senescence-associated marker genes (**Figure 3**). At the same time, the T-DNA insertion mutant *acs7-1* exhibited delayed leaf senescence (**Figure 4**). All of these results further confirmed our aforementioned assumption that ACS7 is one of the major contributors to the synthesis of ‘senescence ethylene.’ However, it should be noted that over-expression of full-length *ACS7* did not induce as severely precocious leaf senescence as expected (**Figure 3**), suggesting post-transcriptional regulation plays more important roles in this process.

As noted above, ACS7 is the only type 3 ACS in *Arabidopsis* and the N-terminus-mediated ACS7 degradation has been suggested to be regulated by both developmental and environmental signals (Xiong et al., 2014). In the present study, we have investigated the effects of senescence signaling on the regulation of ACS7 stability. It was shown that despite a constantly high level of *ACS7* promoter activity as indicated by GUS histochemical staining in the *ACS7:GUS* transgenic *Arabidopsis*, the accumulation of ACS7 protein, i.e., the GUS activities of N^7(1-54)^-GUS, was extremely low in light-grown young seedlings but was gradually restored as plants aging (**Figure 5**). In line with this, even though the transcript and protein accumulation levels of *ACS7* in etiolated *Arabidopsis* over-expressing the full-length or truncated *ACS7* were both remained at approximately the same levels, respectively, ACS7 protein accumulated at a significantly lower level in the young light-grown *35S:ACS7-eGFP* seedlings compared to in their *35S:ACS7*^Δ*1*-*14*^-*eGFP* counterparts (**Figures 6A,B**). Similarly, the transgenic *Arabidopsis* expressing the truncated version of *ACS7* exhibited much more severe precocious leaf senescence (**Figure 6C**). As our previous research has excluded the involvement of light/dark signal in the degradation of ACS7 protein (Xiong et al., 2014), we tempt to speculate that the N-terminus-mediated ACS7 degradation was negatively regulated by senescence signaling. The fact that the transgenic *Arabidopsis* expressing full-length ACS7 also exhibited earlier senescence than the wild-type control implied a recovery of the accumulation of ACS7 protein at the late developmental stages of rosette leaves. This was further corroborated by measurements of the accumulation levels of ACS7 protein in the *35S:ACS7-eGFP* and *35S:ACS7*^Δ*1*-*14*^-*eGFP* plants during leaf development (**Figure 6D**).

We next fused the N-terminal 14 residues of ACS7 to SSPP, a negative senescence regulator previously identified in our research (Xiao et al., 2015). Compared to the *SSPP* over-expressing *Arabidopsis*, the *N*^*7*(*1*-*14*)^-*SSPP* over-expressing seedlings exhibited normal growth rather than restricted rosette sizes at vegetative stages, suggesting that the *N*^*7*(*1*-*14*)^ fragment also exerted a negative effect on the accumulation of SSPP protein. However, after the initiation of leaf senescence, both the *SSPP* and *N*^*7*(*1*-*14*)^-*SSPP* over-expressing plants exhibited significant delays in leaf senescence, as well as remarkable down-regulations of several critical senescence-associated marker genes (**Figures 7, 8**). This is consistent with the western blot results that SSPP accumulation was significantly increased in the senescing *35S:N*^*7*(*1*-*14*)^-*SSPP* plants as shown in **Figure 7B**. All these observations strongly support the idea that the N-terminus-mediated protein degradation is tightly controlled by development and/or senescence signals, independently of ACS7.

Leaf senescence is a complex and highly regulated process that involves degradation of macromolecules such as nucleic acids, proteins, lipids and so on (Lim and Nam, 2007; Park et al., 2007; Morita et al., 2009). Modulation of the protein stabilities of key regulators is one important mechanism to ensure normal leaf senescence process. For instance, enhanced stabilities of thylakoid membrane proteins were observed in a wheat stay-green mutant *tasg1* (Tian et al., 2013). In addition, the stability of transcription factor WRKY53 was reported to be regulated by E3 ubiquitin ligase UPL5 to ensure that senescence is executed in the correct time frame in *Arabidopsis* (Miao and Zentgraf, 2010). Here we show, for the first time, that the stability of an ACS family member, specifically ACS7, can be regulated by senescence signaling to allow optimal ethylene production during leaf development.

It is also noteworthy that *ACS7* may not be the only senescence-associated member of this family present in *Arabidopsis*. As discussed above, several other *ACS*s have also been observed to be up-regulated in both *SARK*-induced and natural leaf senescence. However, whether, or not, these ACSs merely facilitate ACS7-mediated ethylene biosynthesis by forming active dimers with ACS7 or perform additional unique roles in leaf senescence will require further research. The functions of other ACS members that form inactive dimers with ACS7 and exhibit down-regulated expressions during leaf senescence also remain to be clarified. Previous work has shown that loss of *ACS6* in maize, a possible type 3 *ACS*, resulted in a reduction of up to 90% of ethylene production and led to a substantial delay in leaf senescence. At the same time, loss of another member *ACS2* resulted in an up to 45% reduction in foliar ethylene emissions and a moderate delay in leaf senescence (Young et al., 2004). It is clear that the mechanisms underlying post-transcriptional regulation vary among different ACS members (Lee et al., 2017), additional research on the roles of senescence signaling in the post-transcriptional regulation of other ACSs will provide more insights on the regulatory mechanisms underlying ACS turnover during leaf senescence.

## Author Contributions

NNW conceived and designed the study, supervised the experiments, and compiled and finalized the article. GS, DD, LX, LS, XZ, ZW, SL, XY, N, and DW performed the experiments. NNW and YM analyzed the data. YM drafted and wrote the manuscript. NNW drafted and revised the manuscript. All authors read and approved the final manuscript.

## Conflict of Interest Statement

The authors declare that the research was conducted in the absence of any commercial or financial relationships that could be construed as a potential conflict of interest.

## Supplementary Material

FIGURE S1 Southern blot analysis reveals a single-copy T-DNA insertion in the *acs7-1* genome. **(A)** Schematic map of the binary vector pGKB5 used in generation of the *acs7-1* T-DNA insertion mutant. GUS, beta-glucuronidase gene; nos ter, nopaline synthase terminator; ocs Ter, ocs terminator; nptII, kanamycin resistance gene; nor Pro, nopaline synthase promoter; 35S Pro, 35S promoter; Bar, phosphiothrici resistance gene; g7 Ter, g7 terminator. LB and RB, left border and right border. Positions of four restriction enzymes, *Eco*R V (V), *Eco*R I (I), *Pst* I (P), and *Bst*P I (B), were indicated with dark short vertical lines. Position of the probe in the right border was labeled by dark line below the structure. The arrows indicate direction of the transcription. **(B)** Southern blot analysis of the *acs7-1* and WT genomic DNA that were digested with the indicated four different restriction enzymes and hybridized with the right border probe. Primers used to amplify the probe sequence were listed in Supplementary Table S1. Red arrows pointed the hybridized bands in each case in comparison to the wild-type control. The DIG-labeled DNA marker was on the leftmost lane (M).

FIGURE S2 Determination of the transcription levels of *ACS7* in the *GVG:ACS7-Flag* and *GVG:ACS7*^Δ*1*-*14*^-*Flag* transgenic *Arabidopsis*. The 9-day-old transgenic seedlings of *GVG:ACS7-Flag* and *GVG:ACS7*^Δ*1*-*14*^-*Flag* were treated with 10 μM DEX (+DEX) or its solvent, ethanol (mock) for 24 h, and then harvested for RNA extraction and subsequent quantitative RT-PCR analysis of *ACS7* transgene. *TIP41-like* was used as an internal control. All primers used were listed in Supplementary Table S1. Data presented were the typical results from three biological replicates with at least three technical repeats. Error bars represent SD.

FIGURE S3 Effects of *ACS7* loss-of-function mutation on rosette development **(A)**, plant height **(B)**, flowering time **(C)** and blooming period **(D)** of the *Arabidopsis* plants. **(A)** The rosette sizes of *acs7-1* mutant and its wild-type control (WT) were recorded with time. **(B)** The heights of both *acs7-1* and WT were measured at 44 DAE. **(C)** The flowering time was recorded for both *acs7-1* and WT. **(D)** The blooming period was calculated for both *acs7-1* and WT. Bars represent means ± SD. The experiments were repeated at least three times to give the typical results shown here. Asterisk indicates statistically significant differences in student’s *t*-test (α = 0.05).

FIGURE S4 Quantitative RT-PCR analysis of *GUS* expressions in the *ACS7:GUS* and *ACS7:N*^*7*(*1*-*54*)^-*GUS* transgenic *Arabidopsis*. The 9-day-old transgenic seedlings of *ACS7:N*^*7*(*1*-*54*)^-*GUS* and *ACS7:GUS* were harvested for RNA extraction and subsequent quantitative RT-PCR analysis of *GUS*. *TIP41-like* was used as an internal control. **(A)** The comparison on GUS expressions in the transgenic etiolated seedlings of *ACS7:GUS* and *ACS7:N*^*7*(*1*-*54*)^-*GUS*. **(B)** The comparison on GUS expressions in the transgenic light-grown seedlings of *ACS7:GUS* and *ACS7:N*^*7*(*1*-*54*)^-*GUS*. Three biological replicates with at least three technical repeats were performed. Error bars represent SE.

FIGURE S5 Determination of the transcription levels of *SSPP* in the *35S:SSPP-HA* and *35S:N*^*7*(*1*-*14*)^-*SSPP-HA* transgenic *Arabidopsis*. The 9-day-old seedlings of *35S:SSPP-HA* and *35S:N*^*7*(*1*-*14*)^-*SSPP-HA* were harvested for RNA extraction and subsequent quantitative RT-PCR analysis of *SSPP*. *TIP41-like* was used as an internal control. Three biological replicates with at least three technical repeats were performed in each case. Error bars represent SE.

FIGURE S6 The original images of the grouped blots in **Figures 3, 6**. **(A)** The original blot image shown in **Figure 3C** indicating accumulation levels of ACS7-Flag and ACS7^Δ^*^1-14^*-Flag proteins in 25-day-old *GVG:GUS, GVG:ACS7*^Δ*1*-*14*^-*Flag* and *GVG:ACS7-Flag* transgenic *Arabidopsis* plants sprayed with either 30 μM DEX (+DEX) or its solvent, ethanol (mock). **(B)** The original images of grouped anti-GFP and anti-actin blots shown in **Figure 6B** for the detection of ACS7 protein accumulations in etiolated seedlings and 9-day-old light-grown seedlings of both *35S:ACS7-eGFP* and *35S:ACS7*^Δ*1*-*14*^-*eGFP*.
