# Supplementary figures and images for: N-Terminus-Mediated Degradation of ACS7 Is Negatively Regulated by Senescence Signaling to Allow Optimal Ethylene Production during Leaf Development in Arabidopsis
Source: Front Plant Sci. 2017 Dec 6;8:2066. doi: 10.3389/fpls.2017.02066 (PMC5723933; doi:10.3389/fpls.2017.02066)

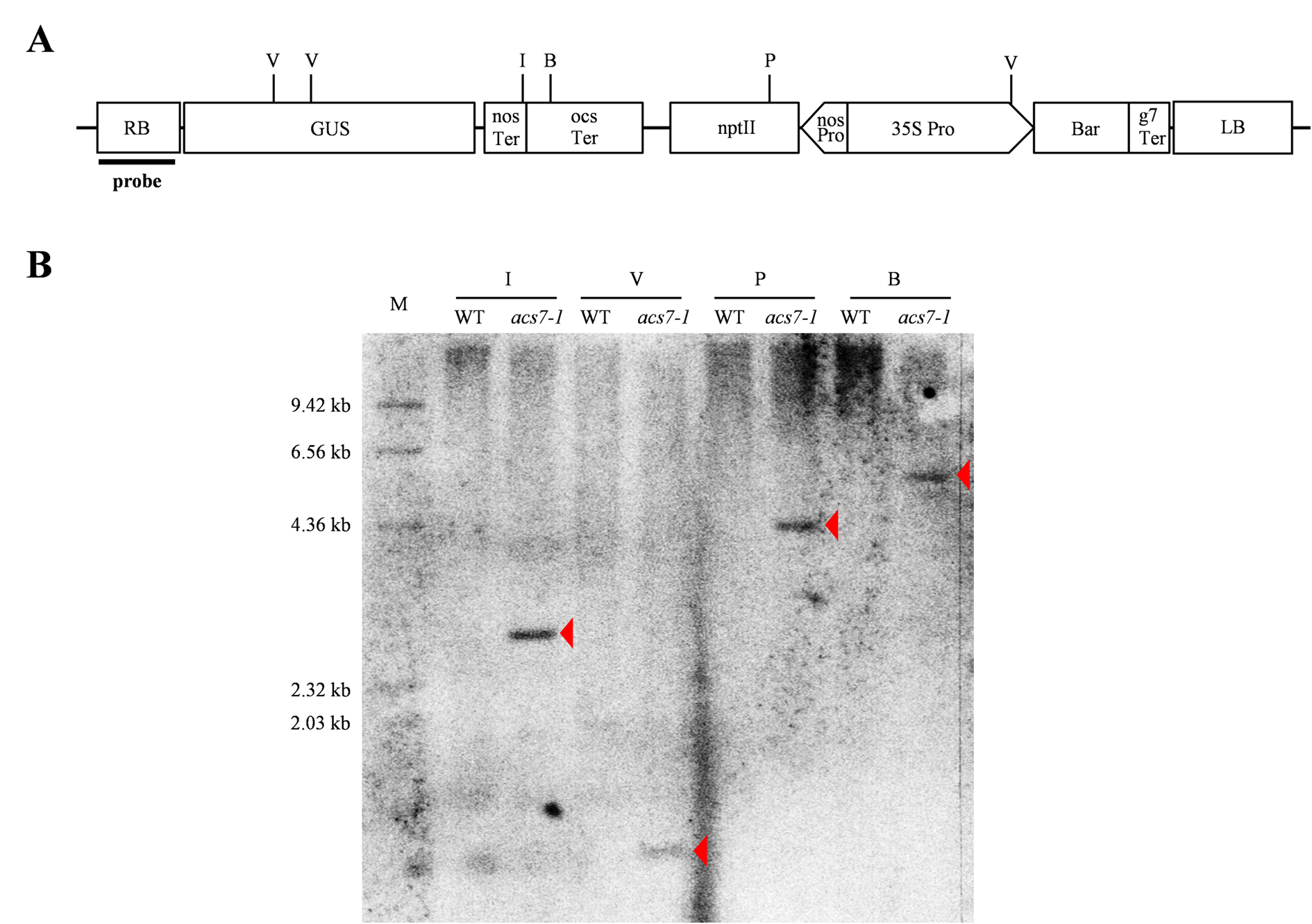

Supplement: FIGURE S1 — Southern blot analysis reveals a single-copy T-DNA insertion in the acs7-1 genome. (A) Schematic map of the binary vector pGKB5 used in generation of the acs7-1 T-DNA insertion mutant. GUS, beta-glucuronidase gene; nos ter, nopaline synthase terminator; ocs Ter, ocs terminator; nptII, kanamycin resistance gene; nor Pro, nopaline synthase promoter; 35S Pro, 35S promoter; Bar, phosphiothrici resistance gene; g7 Ter, g7 terminator. LB and RB, left border and right border. Positions of four restriction enzymes, EcoR V (V), EcoR I (I), Pst I (P), and BstP I (B), were indicated with dark short vertical lines. Position of the probe in the right border was labeled by dark line below the structure. The arrows indicate direction of the transcription. (B) Southern blot analysis of the acs7-1 and WT genomic DNA that were digested with the indicated four different restriction enzymes and hybridized with the right border probe. Primers used to amplify the probe sequence were listed in Supplementary Table S1. Red arrows pointed the hybridized bands in each case in comparison to the wild-type control. The DIG-labeled DNA marker was on the leftmost lane (M). [file Image_1.TIF]

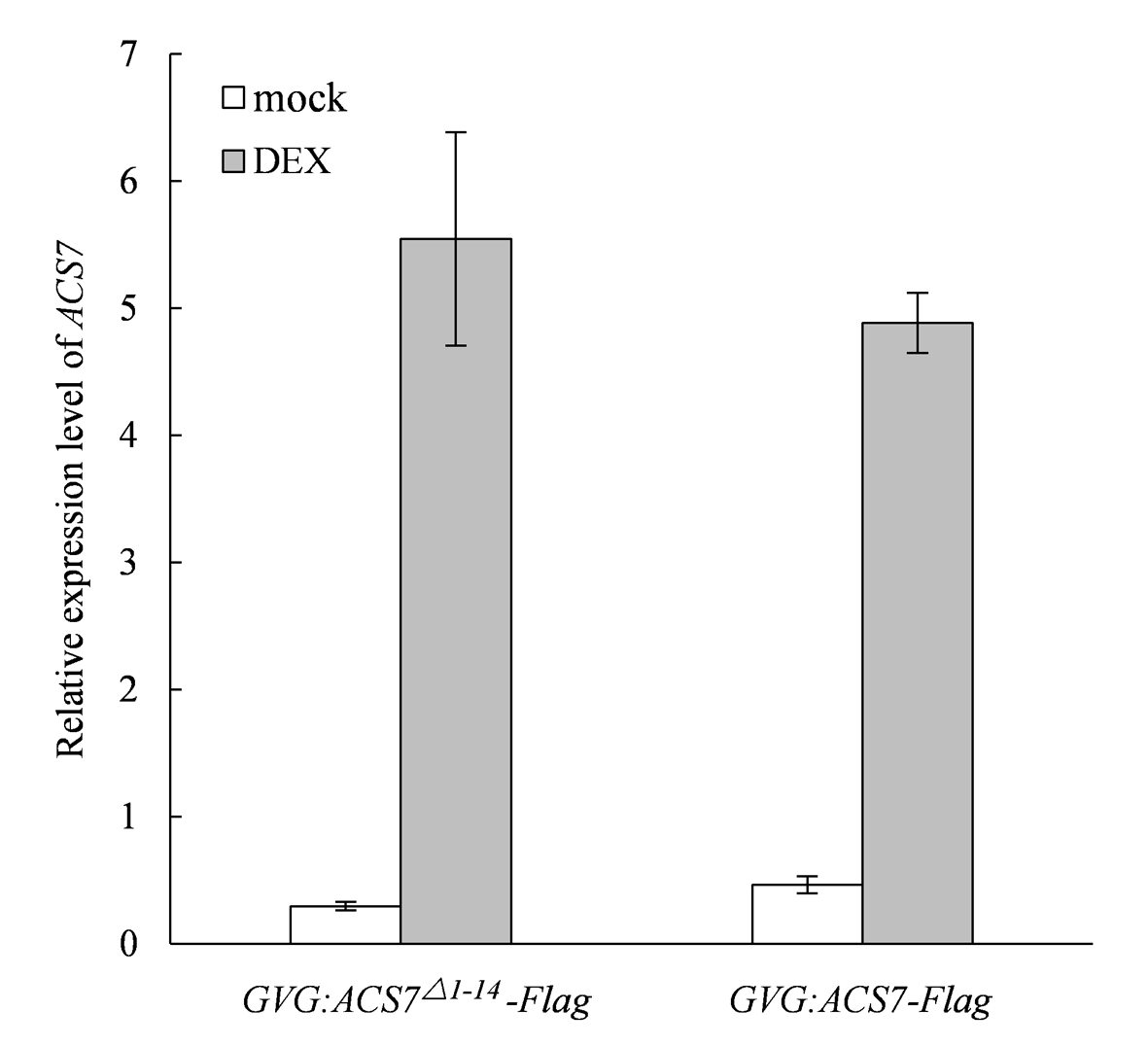

Supplement: FIGURE S2 — Determination of the transcription levels of ACS7 in the GVG:ACS7-Flag and GVG:ACS7Δ1-14-Flag transgenic Arabidopsis. The 9-day-old transgenic seedlings of GVG:ACS7-Flag and GVG:ACS7Δ1-14-Flag were treated with 10 μM DEX (+DEX) or its solvent, ethanol (mock) for 24 h, and then harvested for RNA extraction and subsequent quantitative RT-PCR analysis of ACS7 transgene. TIP41-like was used as an internal control. All primers used were listed in Supplementary Table S1. Data presented were the typical results from three biological replicates with at least three technical repeats. Error bars represent SD. [file Image_2.TIF]

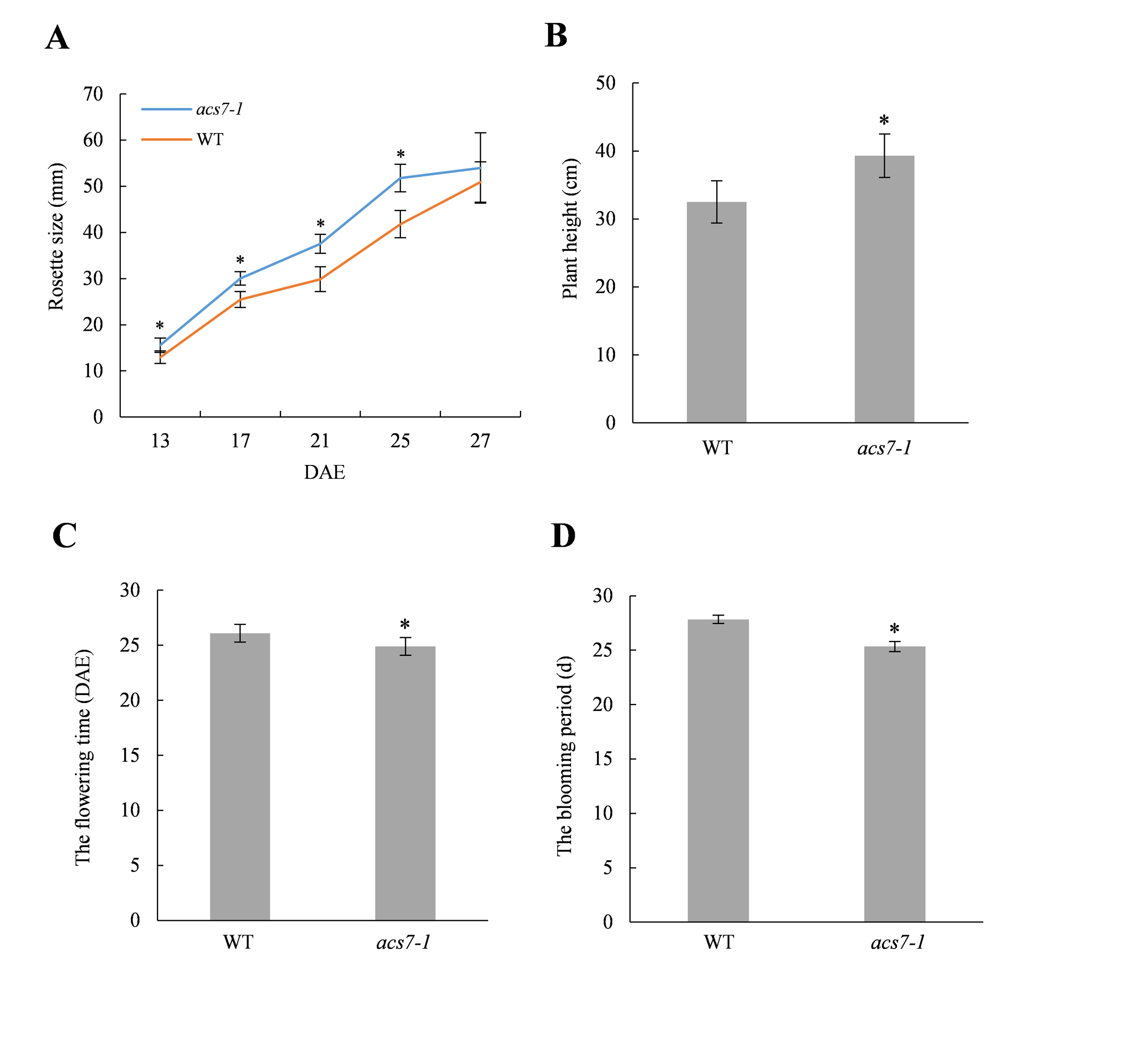

Supplement: FIGURE S3 — Effects of ACS7 loss-of-function mutation on rosette development (A), plant height (B), flowering time (C) and blooming period (D) of the Arabidopsis plants. (A) The rosette sizes of acs7-1 mutant and its wild-type control (WT) were recorded with time. (B) The heights of both acs7-1 and WT were measured at 44 DAE. (C) The flowering time was recorded for both acs7-1 and WT. (D) The blooming period was calculated for both acs7-1 and WT. Bars represent means ± SD. The experiments were repeated at least three times to give the typical results shown here. Asterisk indicates statistically significant differences in student’s t-test (α = 0.05). [file Image_3.TIF]

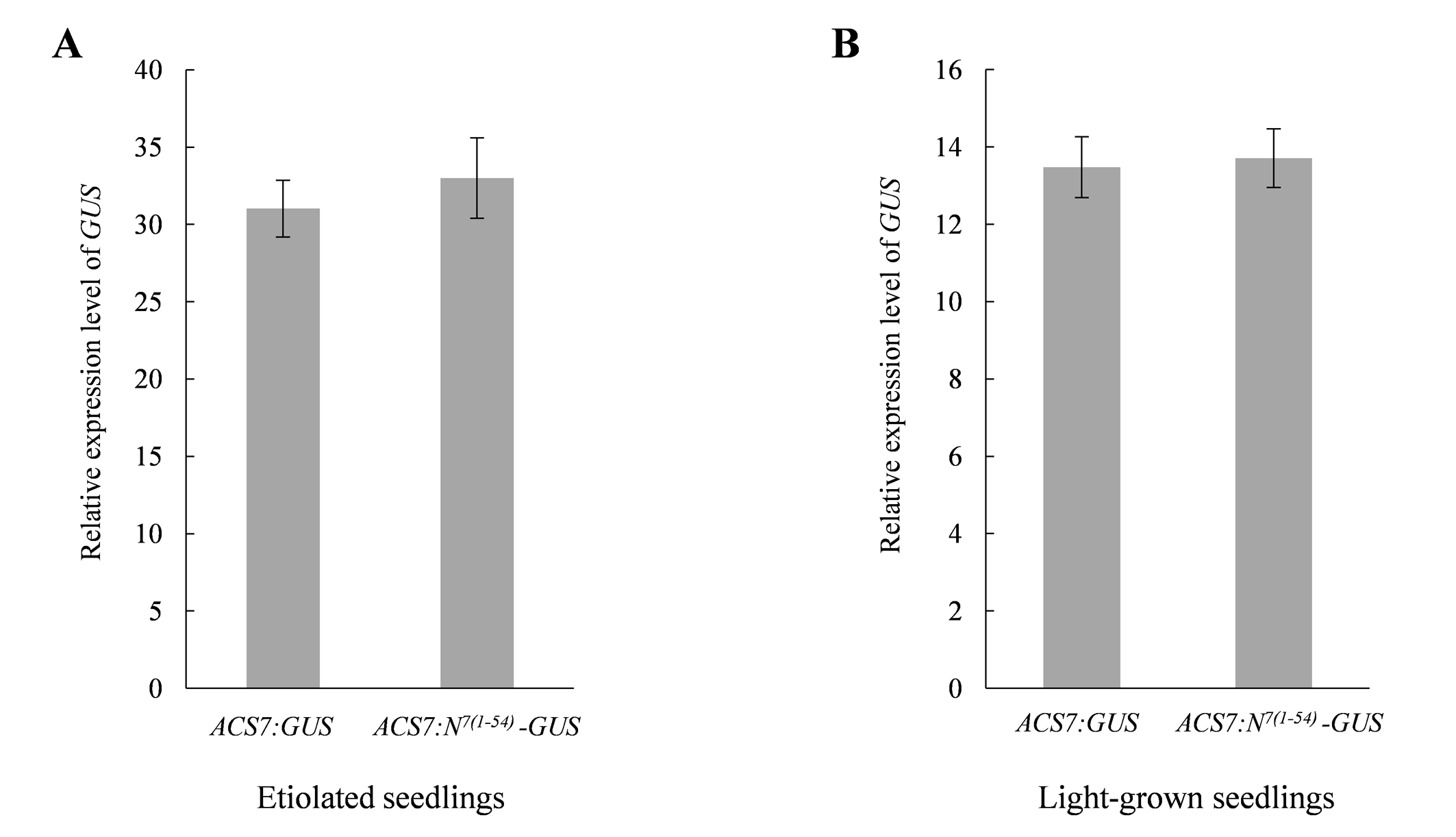

Supplement: FIGURE S4 — Quantitative RT-PCR analysis of GUS expressions in the ACS7:GUS and ACS7:N7(1-54)-GUS transgenic Arabidopsis. The 9-day-old transgenic seedlings of ACS7:N7(1-54)-GUS and ACS7:GUS were harvested for RNA extraction and subsequent quantitative RT-PCR analysis of GUS. TIP41-like was used as an internal control. (A) The comparison on GUS expressions in the transgenic etiolated seedlings of ACS7:GUS and ACS7:N7(1-54)-GUS. (B) The comparison on GUS expressions in the transgenic light-grown seedlings of ACS7:GUS and ACS7:N7(1-54)-GUS. Three biological replicates with at least three technical repeats were performed. Error bars represent SE. [file Image_4.TIF]

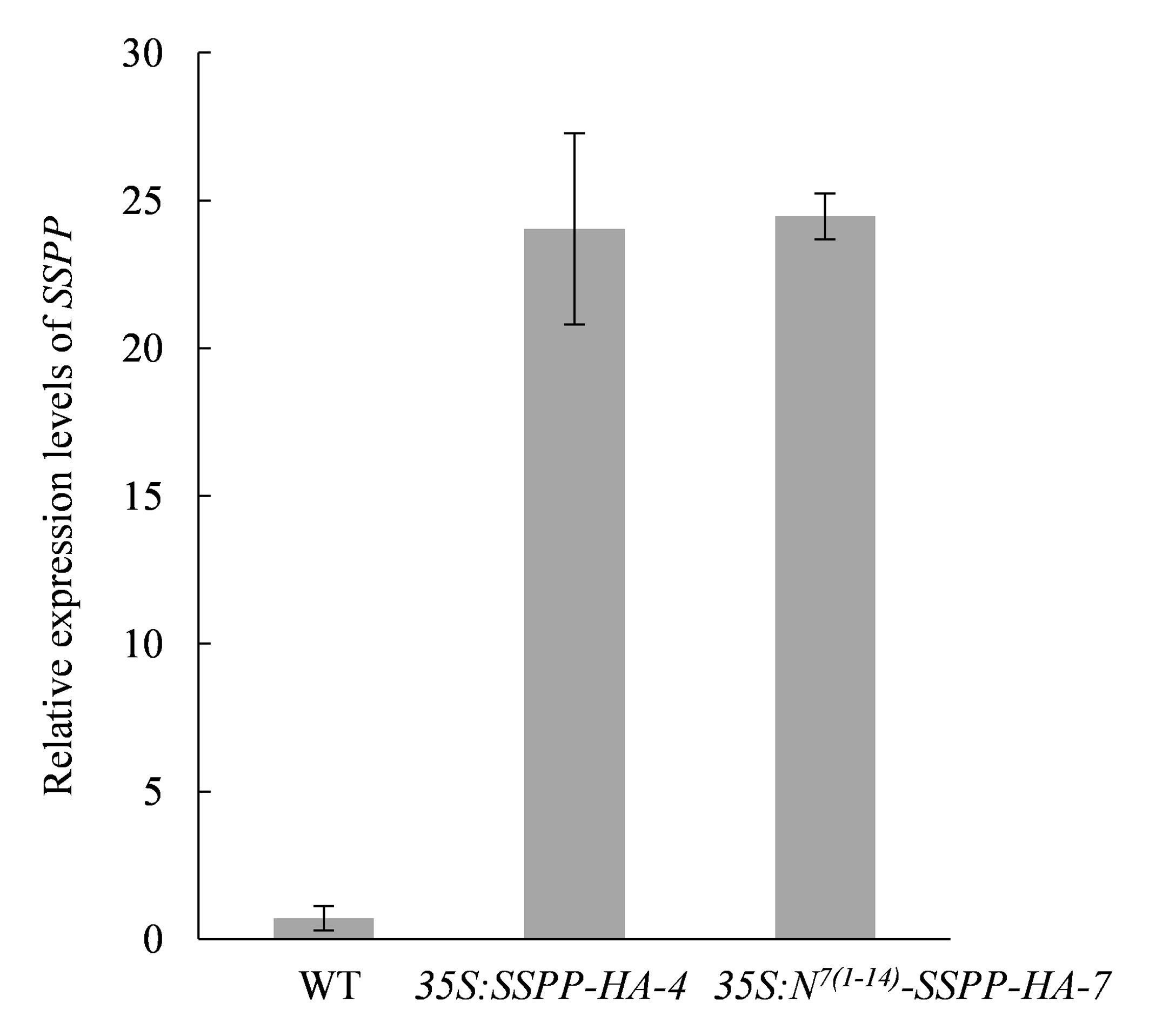

Supplement: FIGURE S5 — Determination of the transcription levels of SSPP in the 35S:SSPP-HA and 35S:N7(1-14)-SSPP-HA transgenic Arabidopsis. The 9-day-old seedlings of 35S:SSPP-HA and 35S:N7(1-14)-SSPP-HA were harvested for RNA extraction and subsequent quantitative RT-PCR analysis of SSPP. TIP41-like was used as an internal control. Three biological replicates with at least three technical repeats were performed in each case. Error bars represent SE. [file Image_5.TIF]

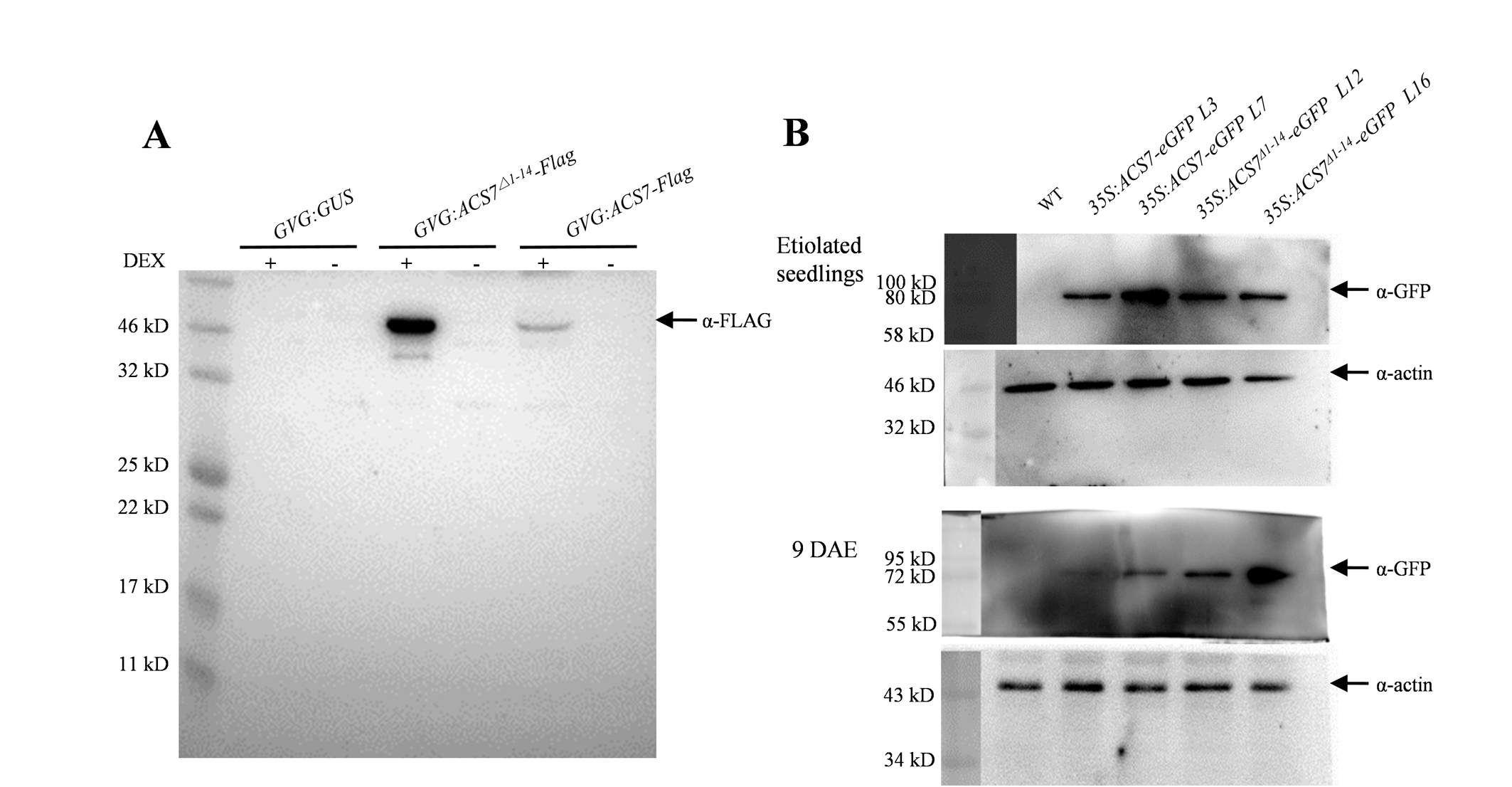

Supplement: FIGURE S6 — The original images of the grouped blots in Figures 3, 6. (A) The original blot image shown in Figure 3C indicating accumulation levels of ACS7-Flag and ACS7Δ1-14-Flag proteins in 25-day-old GVG:GUS, GVG:ACS7Δ1-14-Flag and GVG:ACS7-Flag transgenic Arabidopsis plants sprayed with either 30 μM DEX (+DEX) or its solvent, ethanol (mock). (B) The original images of grouped anti-GFP and anti-actin blots shown in Figure 6B for the detection of ACS7 protein accumulations in etiolated seedlings and 9-day-old light-grown seedlings of both 35S:ACS7-eGFP and 35S:ACS7Δ1-14-eGFP. [file Image_6.TIF]
