# Supplementary material for: N-Terminus-Mediated Degradation of ACS7 Is Negatively Regulated by Senescence Signaling to Allow Optimal Ethylene Production during Leaf Development in Arabidopsis
Source: Front Plant Sci. 2017 Dec 6;8:2066. doi: 10.3389/fpls.2017.02066 (PMC5723933; doi:10.3389/fpls.2017.02066)
Supplement: Supplementary file 7 [file Table_1.docx]

**Supplemental Table 1: Primers used in this study.**

All primers in the current study for quantitative RT-PCR and construct generations are summarized in the following table. The underlined sequences in primers indicate introduced restriction enzyme sites.

| **Primers** | **Sequence (5' to 3')** | **Purpose** |
| --- | --- | --- |
| *ACS7-1* | *TCGTGACGCGAACATTAGAG* | Quantitative RT-PCR of *ACS7* |
| *ACS7-2* | *TCTAGAACCTTCTTTTGGACC* | Quantitative RT-PCR of *ACS7* |
| *RT-GUS-F* | *GGTAGATCTGAGGAACCGACGACTC* | Quantitative RT-PCR of *GUS* |
| *RT-GUS-R* | *GCGATCCAGACTGAATGCCCACA* | Quantitative RT-PCR of *GUS* |
| *rtNAC1-1* | *ACATCCCAAAAATGGCATGC* | Quantitative RT-PCR of *NAC1* |
| *rtNAC1-2* | *TGCTCGGTTAGTTCTCAGCC* | Quantitative RT-PCR of *NAC1* |
| *rtAtNAP-1* | *TTACATGGGACCCGTCTCTC* | Quantitative RT-PCR of *AtNAP* |
| *rtAtNAP-2* | *CCGAACCAACTAGACTCCGA* | Quantitative RT-PCR of *AtNAP* |
| *rtWRKY6-1* | *CAGTTCTCTGGTGGCTCTCC* | Quantitative RT-PCR of *WRKY6* |
| *rtWRKY6-2* | *GTCAGCTGTGAGTGCCGTTA* | Quantitative RT-PCR of *WRKY6* |
| *SAG113-F* | *AACTGCATGTAGCGTCGTTC* | Quantitative RT-PCR of *SAG113* |
| *SAG113-R* | *CTGGCAAATCTCCTCCTCCA* | Quantitative RT-PCR of *SAG113* |
| *rtSSPP-1* | *TGTGATGGAGGATCAGTGCCAGA* | Quantitative RT-PCR of *SSPP* |
| *rtSSPP-2* | *GGAAGCCTCTGGACCTCCATGG* | Quantitative RT-PCR of *SSPP* |
| *TIP41-LIKE-F* | *GTATGAAGATGAACTGGCTGACAAT* | Quantitative RT-PCR of *TIP41-like* |
| *TIP41-LIKE-R* | *ATCAACTCTCAGCCAAAATCGCAAG* | Quantitative RT-PCR of *TIP41-like* |
| *ACS7-eGFP-R* | *GGTGACCTTAAAGCTTCTCGAGCCCGGGGAATTC* | Generation of *ACS7-eGFP,*  *ACS7^Δ1-14^-eGFP* |
| *Δ1-14-eGFP-F* | *GGATCCATGAACGTCGAGCTTTCTCGAGTGG* | Generation of *ACS7^Δ1-14^-eGFP* |
| *35S-N7-SSPP-F1* | *TCTAGAATGGGTCTTCCTCTAATGATGGAGAGATCATCA*  *AACAACAACACTAGT* | Generation of *N^7(1-14)^-SSPP* |
| *35S-N7-SSPP-F2* | *GAGAGATCATCAAACAACAACACTAGTATGGTTAAACCC*  *TGTTGGAGAATAGG* | Generation of *N^7(1-14)^-SSPP* |
| *35S-N7-SSPP-R1* | *GAGCTCTCAAGCGTAATCTGGAACATCGTATGGGTAC* | Generation of *N^7(1-14)^-SSPP* |
| *35S-N7-SSPP-R2* | *AACATCGTATGGGTACTCGAGTGATGTTGAATGCATCGGGTATC* | Generation of *N^7(1-14)^-SSPP* |
| *rtSAG12-1* | *GGATGTCCCGGTTAATGATG* | Quantitative RT-PCR of *SAG12* |
| *rtSAG12-2* | *TGGAAATCAAAACCACCTCC* | Quantitative RT-PCR of *SAG12* |
| *ACS7Flag-rt-F* | *TGCTCGGAGGTCGGATGGTT* | Quantitative RT-PCR of Flag-tagged *ACS7* |
| *Flag-R* | *TGTCGTCATCGTCTTTGTAGTCATCG* | Quantitative RT-PCR of Flag-tagged *ACS7* |
| *pGKB5 RB-F1* | *CTCGTTCTCCTTGGAGTTCTTCG* | Generation of right border probe |
| *pGKB5 RB-R* | *CGTGTGACAGGATATATCTTGTGG* | Generation of right border probe |
